# SLRanger: an integrated approach for spliced leader detection and operon prediction using long RNA reads

**DOI:** 10.1093/bib/bbaf437

**Published:** 2025-09-04

**Authors:** Yanwen Shao, Zhihao Guo, Jinpeng Chen, Runsheng Li

**Affiliations:** Department of Infectious Diseases and Public Health, Jockey Club College of Veterinary Medicine and Life Sciences, City University of Hong Kong, Tat Chee Avenue, Kowloon, Hong Kong, China; Department of Infectious Diseases and Public Health, Jockey Club College of Veterinary Medicine and Life Sciences, City University of Hong Kong, Tat Chee Avenue, Kowloon, Hong Kong, China; Department of Computer Science, College of Computing, City University of Hong Kong, Tat Chee Avenue, Kowloon, Hong Kong, China; Department of Infectious Diseases and Public Health, Jockey Club College of Veterinary Medicine and Life Sciences, City University of Hong Kong, Tat Chee Avenue, Kowloon, Hong Kong, China; Tung Biomedical Sciences Centre, City University of Hong Kong, Tat Chee Avenue, Kowloon, Hong Kong, China; Department of Precision Diagnostic and Therapeutic Technology, City University of Hong Kong Matter Science Research Institute (Futian) Shenzhen, 3 Binglang Road, Fubao Street, Futian District, Shenzhen, Guangdong, 518045, China

**Keywords:** spliced leader trans-splicing, eukaryotic operon, genome annotation, long-read RNA

## Abstract

Spliced leader (SL) trans-splicing occurs in a wide range of eukaryotes and plays a critical role in processing mRNAs derived from operon structures. However, current research on this mechanism remains limited, partly due to the difficulty in accurately identifying genuine SL trans-splicing events. The advent of long-read RNA sequencing technologies, such as direct RNA sequencing by Oxford Nanopore Technologies, offers a more promising avenue for detecting these events with greater resolution. Here, we present SLRanger, an integrated tool to detect SL sequences and predict operon structures in eukaryotic transcriptomes. SLRanger improves upon the traditional Smith–Waterman (SW) alignment framework by incorporating an optimized scoring scheme tailored to SL detection in native long RNA reads. We primarily validated our method using direct RNA sequencing data from *Caenorhabditis elegans*, a well-established model organism for studying trans-splicing. Through a dynamic cutoff strategy, SLRanger robustly identified high-confidence SL-carrying reads. Leveraging the SL information, SLRanger achieved over 80% accuracy in operon gene prediction, recovering more than 70% of known operon genes in *C. elegans*. SLRanger was also applied to detect SL from cDNA long RNA reads and another trans-spliced species. Our results demonstrate that SLRanger not only provides a reliable approach for characterizing SL trans-splicing events but also serves as an effective framework for operon discovery, enabling transcriptomic analysis for operons and facilitating downstream data-mining applications.

## Introduction

Spliced leader (SL) trans-splicing is a unique RNA processing mechanism in which a short and conserved RNA fragment (namely SL) is added to the 5′ end of precursor messenger RNAs (mRNAs) [1–3]. Trans-splicing is extremely rare and confined to intragenic events in vertebrates [4–6]. However, in a diverse set of other eukaryotes, it is a genome-scale phenomenon involving dedicated cellular machinery. Taxa with frequent trans-splicing include nematodes, kinetoplastid parasites, such as *Trypanosoma*, flatworms, cnidarians, and some tunicates [7–9]. Among these species, SL trans-splicing serves as the predominant mechanism for removing the 5′ outron from pre-mRNA, thus critical to RNA stability, and translation efficiency [10–12]. One clear biological function of SL trans-splicing is processing polycistronic transcripts derived from eukaryotic operons into monocistronic mRNAs, facilitating proper mRNA processing and gene regulation. In nematodes such as the model organism *Caenorhabditis elegans*, around 70% of transcripts are processed by trans-splicing, and the mature transcript contains an SL after processing. Notably, trans-splicing in nematodes is mediated by two types of SLs, namely SL1 and SL2 [13–16]. While around 50% of all genes are trans-spliced with SL1, more than 17% of transcripts are processed via SL2-mediated trans-splicing, which is strongly associated with operon-derived transcripts and reflects the underlying operon organization [16, 17].

Although the concept of SL trans-splicing in eukaryotic organisms has been proposed for decades [18, 19], its biological significance and functional roles remain incompletely understood [20, 21]. A major bottleneck in this field is the difficulty in systematically identifying SL trans-splicing events. Even for the model organism like *C. elegans*, the current genome release did not include a comprehensive SL annotation for each protein-coding gene [22]. A detailed annotation for SL in each gene is a hard job for several reasons. SL sequences are not located immediately upstream of the transcription start site (TSS) of each gene on the genome. When the full transcript is aligned back to the genome, the SL sequence will be presented as an “unaligned end.” Secondly, the SL sequence on mature mRNA is extremely short, from ~22 nt in *C. elegans* to ~35 nt in *T. brucei* [23]. Moreover, since most of the modern high-throughput RNA-seq techniques rely on poly(A) tail selection to enrich mRNAs, they will only ensure the intact sequencing of the 3′ end of the transcript. As a result, the SL sequence in the 5′ end could be partially or entirely missed due to the mRNA degradation [24]. Thirdly, in nematodes, the sequence similarity among different SL types (e.g., SL1 vs. SL2 in *C. elegans*) is high [25], making it hard to distinguish SL from each other without a fully recovered sequence. Therefore, a specialized method capable of accurately detecting and annotating SL sequences is urgently needed to overcome these limitations.

Current studies on eukaryotes harboring SL structures predominantly rely on next-generation sequencing (NGS) technologies, which yield short reads of only about 150–250 base pairs. However, such fragmented RNA reads often hinder the identification of full-length SL sequences and the resolution of operon structures, thereby limiting a comprehensive genome-wide analysis of SL usage [26]. Existing tools using short-read RNA sequencing without SL-specific amplification have not satisfactorily detected SLs and operons, even in the model organism *C. elegans* [26–28]. Moreover, short reads couldn’t easily distinguish between similar SL sequences or map them to repetitive SL RNA gene loci. Wenzel *et al*. [26] have developed SL finding and operon detection pipelines for short reads, but can only achieve 19% sensitivity in *C. elegans*. Some researchers employ PCR amplification of SL sequences to generate large numbers of complete SL sequences [29–31] to facilitate the detection of SL trans-splicing events and the prediction of operons. However, PCR amplification only guarantees that the amplified SL type can be found and can’t display the actual abundance of each trans-spliced gene or operon.

The advent of third-generation long-read sequencing technologies, including Oxford Nanopore Technologies (ONT), has enabled the direct sequencing of full-length RNA molecules. This advancement increases the possibility of accurately identifying complete transcript structures [32, 33]. The SL sequences at the 5′ ends of transcripts could also be sequenced with the full-length transcript, which could be directly assigned to the gene of origin. Despite the high similarity between SL1 and SL2 sequences, the difference between these sequences could help distinguish the RNA reads containing SL1 or SL2 sequences. Consequently, these long reads present a unique opportunity to improve SL detection and operon prediction. Bernard *et al*. [31] detected SLs and discovered the mimic hairpin structure of non-trans-spliced transcripts in *C. elegans* using ONT cDNA data. However, long-read RNA sequencing also introduces new challenges. An unneglectable issue is a relatively high error rate compared to NGS short reads [34, 35]. Reads from nanopore RNA002 technique have an average read accuracy of 95%, and the 5′ ends of long reads are prone to even higher rates of errors [32, 36, 37]. Combined with the frequent occurrence of 5′ truncation, detecting SL sequences using third-generation sequencing remains highly challenging.

A robust and comprehensive scoring system is essential to fully leverage the advantages of third-generation sequencing while mitigating its limitations. Such a system must distinguish true SL sequences from sequencing artifacts, account for short and error-prone alignments, and ultimately enhance the reliability of SL identification and operon prediction from long RNA reads. Here, we propose SLRanger, an integrated approach specifically designed to address these needs, which could further advance the study of eukaryotic trans-splicing and operon regulation. SLRanger provides a scoring system to detect SLs for long-read RNA and consequently predict operon structure.

## Materials and methods

**Figure 1:**
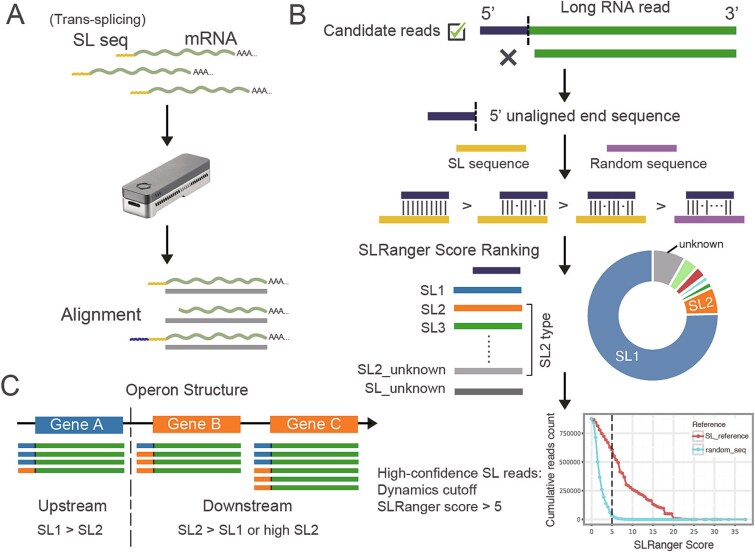
Workflow of SLRanger. (a) The direct RNA sequencing workflow for species exhibiting trans-splicing. In such species, spliced leader (SL) sequences are located at the 5′ ends of RNA reads due to the trans-splicing mechanism. Mature mRNAs containing SL sequences are sequenced using Oxford Nanopore direct RNA sequencing, and the resulting long reads are subsequently aligned to the reference genome. (b) The SL detection module of SLRanger. After aligning long RNA reads to the reference genome, the unaligned 5′-end fragments are extracted and aligned against a reference set of SL (including SL1 sequence and all SL2 variant sequences) and random sequences (as controls). Using SLRanger’s scoring scheme, each candidate read is assigned an “SLRanger score.” Reads will be assigned to the corresponding SL-type according to the highest “SLRanger score.” Reads that can’t be classified as SL1 will be considered as the SL2 type. If SL1 and SL2 can’t be distinguished, the read is labeled SL_unknown. If variants of SL2 can’t be distinguished, the read is labeled SL2_unknown. A dynamic cutoff is then applied to identify “high-confidence SL reads.” (c) the principle underlying operon prediction by SLRanger. Based on the presence of high-confidence SL sequences in each read and their genomic mapping positions relative to gene annotations, operon structures are inferred. Genes with a high proportion of SL1-type reads are predicted to be upstream operon genes, whereas genes with a high proportion of SL2-type reads or supported by multiple SL2-type reads are predicted to be downstream operon genes.

SLRanger encompasses two primary functions, SL detection (Fig. 1b) and operon prediction (Fig. 1c), used to determine whether long RNA reads carry SL sequences and predict the operon structure based on the SL information.

Our main contributions are as follows. We established a scoring system to identify SLs in long RNA reads, allowing users to select high-confidence SL reads based on the score assigned to each read. Additionally, SLRanger effectively distinguishes between different SL types [14], including SL1 and 12 SL2 variants. We have validated the high accuracy and sensitivity of two functions in SLRanger by verifying the predicted operons with annotated operons.

### Candidate reads preprocessing

The long RNA reads are mapped to the genome reference, and only primary alignments are retained for subsequent processing (Fig. 1a). Because SL trans-splicing is characterized by its specificity at the 5′ end of transcripts, only reads with unaligned soft-clipped sequences (length ≥ 5) at the 5′ end of primary alignments are regarded as candidate reads. The unaligned sequences, namely candidate sequences, were extracted for further processing.

### Scoring system for spliced leader detection

Users should prepare a FASTA file of SL references for the sequenced species, which should be the mature SL sequence occurring in the mature mRNA. For *C. elegans*, we obtained the SL references of SL1 and 12 SL2 variants from a well-established literature [14], which is also provided in the supplementary link. Candidate sequences are mapped to SL references using the SW algorithm (match = 1, mismatch = 1, gap_open = 1, gap_extend = 1), yielding an optimal score (SW score). We have applied several modifications to better detect the SL sequences than the raw SW score. The optimal locally mapped sequence region is used for the following scoring steps. First, we check for a continuous stretch of five bases mapped to the reference with at most one mismatch. A higher occurrence of continuous mapping results in a higher score. Second, the score increases if the locally mapped region is positioned closer to the 3′ end of the reference. Third, the score is higher if the locally mapped region is closer to the 5′ end of the aligned region. Lastly, the resulting final score is normalized by the maximum possible score for the length of the locally mapped region sequence, yielding the “SLRanger score.” The maximum score is calculated using the reference sequence with the same length. Reads will be scored by aligning to sequences of SL1 and all SL2 variants, but only the highest “SLRanger score” and the corresponding SL name will be recorded. Both the occurrence of the SL1 and SL2 sequences can reflect the integrity of mRNA reads. However, SL2-type reads, which are trans-spliced by SL2 sequences, are highly associated with operons. Therefore, to better demonstrate the ability of SLRanger to distinguish between these two SL types, we labeled reads as “unknown” if they were aligned to multiple SL references with identical highest SLRanger scores. Moreover, SL_unknown denotes reads that cannot be definitively classified as either SL1-type or SL2-type, and SL2_unknown denotes reads that cannot be assigned to any specific SL2 variants.

To mitigate randomness, we generated 10 random sequences as controls (generated by the Python function “random”). Each sequence was set to the round mean length of the SL sequences, which will be calculated automatically from the user-provided “reference FASTA” file. In this case, random sequences are 22 nucleotides for *C. elegans*. Candidate sequences are mapped to all the random sequences in the same method as the SL references. The highest “SLRanger scores” of random sequences were also recorded as background. If a candidate read’s highest mapping score from any of the SL sequences exceeds all the random sequences, it is classified as a “potential SL read.” The “potential SL reads” are further used to obtain reads carrying high-confidence SL sequences in the next step.

The SL detection module of SLRanger provides detailed output for each read, including the best score from the SW algorithm (SW score), the SLRanger score, and the SL type.

### Screening reads carrying high-confidence SL sequences

A dynamic cutoff is calculated to select “potential SL reads” carrying high-confidence SL sequences. We defined the reads that meet the dynamic cutoff as “SLRanger high-confidence SL reads” (Fig. 1b). To get the cutoff, we first record the number and score for all reads from the alignment to the SL reference sequences and to a set of random control sequences. Then, we defined the dynamic cutoff based on the fold difference between the number of reads aligned to the SL references and those aligned to random sequences (default = 4, can be modified via option --cutoff), where a higher fold difference indicates a more stringent criterion. Users may fine-tune the dynamic cutoff to retrieve more SL-related information as needed. This process can be formalized as:


(1) \begin{equation*} {s}_c=\min \left\{s\in \mathcal{S}\ |\ \frac{N_{\mathrm{SL}}(s)}{N_{\mathrm{random}}(s)}\ge f\right\} \end{equation*}

where $\min \left\{\bullet \right\}$ returns the smallest score $s$ in $\mathcal{S}$ for which the specified condition ($\frac{N_{\mathrm{SL}}(s)}{N_{\mathrm{random}}(s)}\ge f$) is satisfied. $f$ stands for the fold difference between the number of reads aligned to the SL reference and those aligned to random sequences. $\mathcal{S}$ is the set of all distinct SLRanger scores obtained from the candidate reads, ${N}_{\mathrm{SL}}(s)$ denotes the number of reads with score $s$ that align to the SL reference sequences, ${N}_{\mathrm{random}}(s)$ indicates the number of reads with score $s$ that align to the random control sequences, and ${s}_c$ is the resulting dynamic cutoff, which is used to distinguish high-confidence SL-containing reads from background noise.

**Figure 2:**
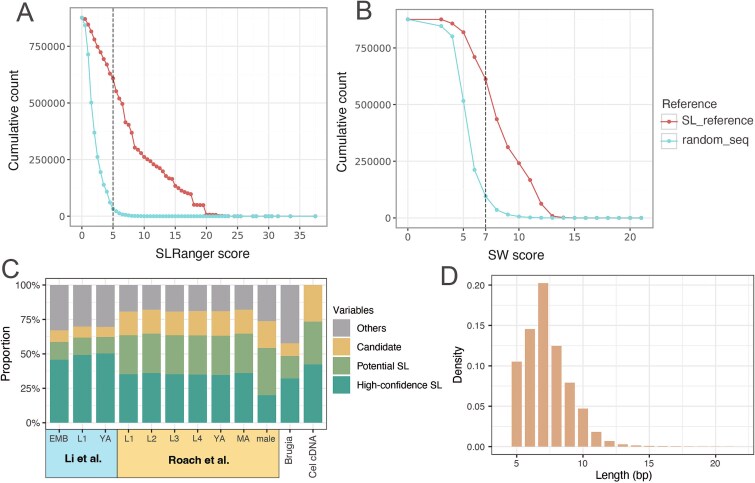
Performance of SLRanger in identifying high-confidence SL-containing reads using the dynamic cutoff. (a and b) AUC plots illustrate the dynamic cutoff performance of SLRanger score (A) and smith-Waterman (SW) alignment optimal score (B) in selecting high-confidence SL reads. The example uses the *C. elegans* embryo dataset from Li *et al*. dashed lines indicate the score (dynamic cutoff) where the number of “potential SL” reads is four times that of “random” reads. Compared to the SW score, the SLRanger score achieves a larger area between the red (potential SL) and blue (random) lines, indicating a higher enrichment of genuine SL reads. (c) Ratio of candidate reads with unaligned 5′ ends versus the number of high-confidence SL reads identified using SLRanger’s dynamic cutoff across all datasets carrying spliced leader. “Other” implies reads with no soft clipping or soft clipping length didn’t reach 5 nt. “Candidate”: If a read with unaligned soft-clipped sequences (length ≥ 5) at the 5′ end of primary alignments is regarded as “candidate reads”; “Potential SL”: Read’s highest mapping score to any of the SL sequences exceeds all the random sequences, it is classified as “potential SL read”; “High-confidence SL”: reads have higher SLRanger score compared to the dynamic cutoff are regarded as carrying high-confidence SL sequences, namely “High-confidence SL read.” (d) Length distribution of SL sequence fragments retained in the unaligned 5′ regions of high-confidence SL reads.

To better visualize the dynamic cutoff selection process, we have provided a visualization function in SLRanger to show the corresponding SLRanger score that meets the cutoff and the number of solid reads identified by the default cutoff (Figs 1b, 2a and b, Supplementary  S2). The *Y*-axis reflects the cumulative count of candidate reads whose score exceeds each threshold. At a score of 0, the number equals the total candidate reads being analyzed. A greater Area Under Curve (AUC) difference between SL and random sequences indicates better discrimination, demonstrating improved sensitivity in identifying true SL-containing reads.

### SLRanger operon prediction

Similar to many existing approaches, our operon prediction method relies on identifying the combinations of SL1 and SL2 spliced leader signals in adjacent genes. According to the definition of an operon in *C. elegans*, the upstream gene (i.e., the first gene in an operon) is typically associated with SL1-type reads, while downstream genes (starting from the second gene onward) are associated with SL2-type reads (Fig. 1c) [16, 26, 38, 39]. Based on this principle, the operon prediction pipeline requires the following inputs: the species’ General Feature Format (GFF) annotation file, the gene assignment of each read (can be obtained via the Trackcluster addgene function [33]), and the SL detection information for each read. Only “SLRanger high-confidence SL reads” that can be clearly distinguished between SL1 and SL2 (excluding those labeled as SL_unknown) are retained for downstream operon detection. We found that a certain number of reads are sufficiently long to span multiple genes (i.e. fusion reads defined in Li *et al*. [33]), which are possible reads that capture the partially processed operonic transcripts. For fusion reads, we modified the addgene function from Trackcluster by applying a stricter threshold to determine the correct gene assignment and reduce false positives. A gene name would be assigned to a read if at least one-third of the exon region from both the read and reference annotation has an intersection.

We first quantify the number of SL1-type and SL2-type reads mapped to each gene. For fusion reads, the SL type is assigned to the first gene and to any downstream genes whose start positions fall within half the length of the first gene. A gene is classified as an upstream gene if it has more SL1-type reads than SL2-type reads; otherwise, it is considered a downstream gene. Additionally, if the proportion of SL2-type reads exceeds one-fourth of the total reads for a gene and over five reads supporting SL2-type, the gene is considered to have both upstream and downstream roles.

Next, using gene coordinates from the GFF annotation, genes are grouped based on the span of their promoter regions (default: 5000 base pairs). Within each group, we identify candidate operons by examining whether adjacent genes exhibit an upstream–downstream pattern, i.e. the first gene is SL1-type and subsequent genes are SL2-type. A gene pair or cluster is confirmed as an operon if at least three reads support the downstream gene(s). Additionally, if a fusion gene is supported by more than three fusion reads and includes an SL2-type gene, this combination is also recognized as an operon. The predicted operons are then output in GFF format.

### Used datasets

Among eukaryotic organisms with operon structures, *C. elegans* is one of the most extensively studied species and has comprehensive publicly available direct RNA sequencing data generated using ONT. Without prior amplification, direct RNA sequencing provides data that represent the actual abundance of transcript variants throughout the transcriptome. Consequently, SLRanger is primarily focused on analyzing direct RNA sequencing datasets of *C. elegans* and using its operon annotations as a positive dataset for validation. SLRanger could also be applied to cDNA data. Nonetheless, users could apply SLRanger in the mode of RNA or cDNA for SL detection and operon prediction according to their own long RNA-seq data.

To demonstrate the capabilities of SLRanger, we analyzed four publicly available datasets from previously published works [32, 33]. For the *C. elegans* direct RNA sequencing dataset from Li *et al*., we performed basecalling on the FAST5 files using the updated Dorado model rna002_70bps_hac@v3, which is optimized for RNA002 chemistry (available at: https://github.com/nanoporetech/dorado). The resulting FASTQ files were also deposited in public repositories (PRJNA1254851). Applying the updated basecaller improved the *Q* score and mapping accuracy of the reads, from 7.6 to 14.8 and from 82.7% to 93.2%, respectively (Supplementary Fig. S1). Another *C. elegans* direct RNA sequencing dataset is from Roach *et al*. [32], and we used the FASTQ files deposited in the European Nucleotide Archive (ENA) dataset directly (PRJEB31791). For the different stages in the Roach *et al*. dataset [32], we combined replicates from various developmental stages for downstream analysis with SLRanger, including larva stage 1 (L1), larva stage 2 (L2), larva stage 3 (L3), larva stage 4 (L4), young adult (YA), mature adult, and male. For the cDNA data, we downloaded the FASTQ files deposited in the public repositories (PRJNA822363) from Bernard *et al*. [31]. Average *Q* scores ranged from 7 to 9, and mapping rates from 40% to 90%. Consequently, SRR18584059, which combined the largest yield with the best quality, was used for spliced leader evaluation. In addition to *C. elegans*, we also used direct RNA sequencing data from another species possessing a SL sequence, *Brugia malayi* (PRJNA944578) [40], to evaluate the ability of SLRanger to detect SLs. As there is no ground truth for SL detection, we also processed a human direct RNA dataset lacking SLs to showcase the low false positive rate of SLRanger (PRJNA1135158) [41].

All *C. elegans* FASTQ files were aligned to the *C. elegans* reference genome (WBcel235) in Wormbase [22], using minimap2 v2.17 with the following parameters optimized for spliced alignment of ONT reads: -ax splice -uf -k14 [42]. GFF annotation for *C. elegans* was also downloaded from Wormbase release WS292 [22]. The dataset of *B. malayi* and human species was aligned to their respective reference genome, B_malayi-4.0 and GRCh38.p14, using the same methodology as *C. elegans*.

## Results

### SLRanger accurately identified reads carrying SL sequences

To evaluate the accuracy and efficiency of SLRanger in detecting SL-containing reads, we compared the SL scores generated by SLRanger to the raw scores produced by the SW algorithm, which was originally used in Li *et al*.’s paper [33]. As a representative example, we used the embryo stage from the Li *et al*. dataset (Fig. 2a and b), while a comprehensive comparison across all datasets is presented in Supplementary Fig. S2.

Across all datasets, the separation between the score distributions of true SL sequences and random sequences was consistently more pronounced under the SLRanger scoring system compared to the raw SW scores. Considering that most candidate reads should contain an SL sequence at the 5′ end, a higher proportion of reads mapped to SL sequences than the random sequence would indicate a better distinction in SL finding.

The AUC distinguishing SL and random sequences was 1.8–2-fold higher with SLRanger than with raw SW scores in both direct RNA and cDNA sequencing data of *C. elegans* (Fig. 2a and b). The shape of the random sequence curve differs between SW and SLRanger due to the distinct scoring mechanisms used by the two methods. SLRanger is specifically designed to enhance the discrimination between true SL sequences and random sequences, leading to a sharper decline in scores for the random set. This demonstrated that the modifications from the original SW score in SLRanger provided better sensitivity to distinguish true SL sequences from background noise or non-SL sequences.

For the human species datasets without SLs, when reads were mapped simultaneously to both SLRanger and a random sequence, the two curves showed consistent trends (Supplementary Fig. S2, Human species SRR32418660 (Yerin *et al*.)). As a result, a cutoff to identify SL-containing reads can’t be established, and no reads can be identified as SL reads. This demonstrated how SLRanger can control the false positive rate to a low level. For *B. malayi*, which possesses the same SL1 sequence as *C. elegans* [43], we were also able to successfully identify its SL using the SL1 sequence from *C. elegans*, with the AUC of 2.2 (Supplementary Fig. S2, *B. malayi* SRR23886071 (Kaylee *et al*.)). And 31% high-confidence SL1 reads were detected in the *B. malayi* dataset.

To further assess detection resolution, we derived the dynamic cutoff under both scoring systems and calculated the proportion of “SLRanger high-confidence SL reads.” SLRanger outperformed SW scoring by identifying ~7% to 15% more high-confidence SL reads across all datasets. This improvement confirmed that SLRanger scores were more efficient, resulting in the recovery of more reads carrying high-confidence SL sequences.

For each dataset, we counted candidate reads, potential SL reads, and high-confidence SL reads. Among all reads in each direct RNA sequencing dataset, ~67% to 81% were selected as candidates for SL detection (Fig. 2c). While the proportion of potential SL reads was comparable across datasets, the proportion of high-confidence SL reads was consistently lower in the Roach *et al*. dataset than in the Li *et al*. dataset. This difference may be attributed to the improved basecalling accuracy and longer sequencing length in the Li *et al*. dataset, which was re-processed using the Dorado model. Both the estimated and observed accuracy of RNA reads were improved in Li *et al*. dataset compared to Roach *et al*. dataset (Supplementary Table S1 and Supplementary Fig. S1). Lower sequence accuracy in the soft-clipped regions of Roach *et al*.’s reads may hinder effective separation from random sequences. Users may fine-tune the dynamic cutoff to retrieve more SL-related information as needed.

In the *C. elegans* direct RNA sequencing dataset, we observed that the length of 5′ end soft clipping is predominantly concentrated within 11 nucleotides (Supplementary Fig. S1c). After identifying high-confidence SL reads, we observed that the length distribution of detected SL-containing residual reads at the 5′ end of SL primarily ranges from 5 to 9 nucleotides (Fig. 2c). Due to the nature of the sequencing direction of nanopore direct RNA sequencing, it is common for the 5′ end of reads to be incomplete. This observation underscores the importance of using a specialized tool like SLRanger, which is designed to detect SL sequences using short residual fragments.

### SLRanger effectively distinguished between SL1 and SL2 sequence types in *C. elegans*

In *C. elegans*, there are two major types of SL sequences: SL1 and SL2. It is well-established that among genes with trans-spliced events, ~75% of genes are trans-spliced with SL1 sequences [16]. While SL1 consists of a single conserved sequence, SL2 sequences are diverse, comprising at least 12 known variants [14]. SL2 trans-splicing is strongly associated with operon structures and serves as a key indicator for operon prediction.

**Figure 3:**
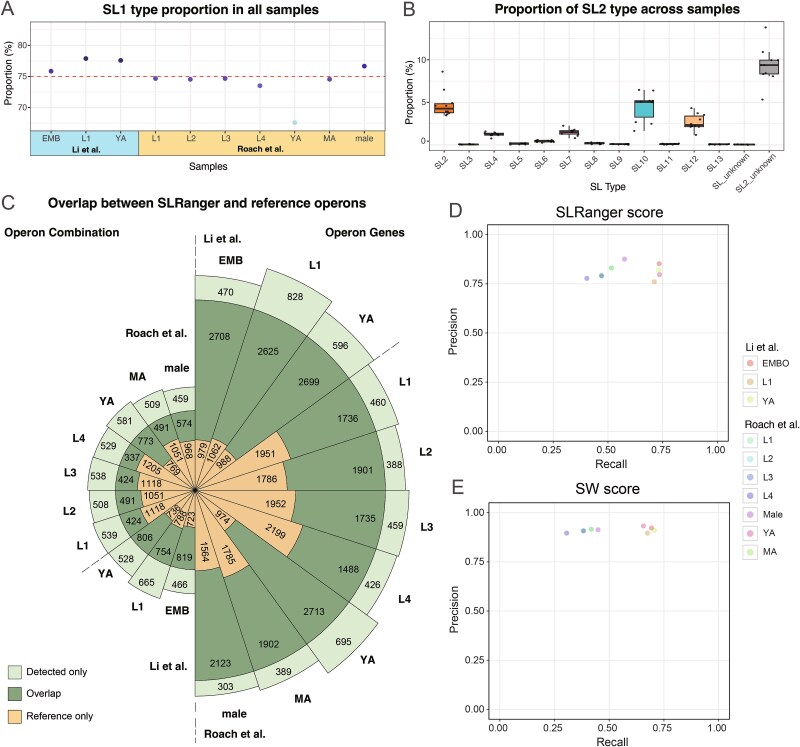
Validation of SL detection and operon prediction of *C. elegans* direct RNA sequencing datasets. (a) The proportion of SL1-type reads across all datasets. (b) The distribution of non-SL1 types across datasets. SL_unknown denotes reads that cannot be definitively classified as either SL1 or SL2, and SL2_unknown denotes reads that cannot be assigned to any specific SL2 variant. (c) Comparison of operons identified by SLRanger with known operon annotations in *C. elegans*. From inner to outer rings, the plot displays: Annotated operons not detected by SLRanger (references only), the intersection between SLRanger predictions and annotated operons (overlap), and operon candidates uniquely predicted by SLRanger but not present in the annotation (detected only). (d and e) The recall and precision result of operon prediction by SLRanger (d) and SW (e) high-confidence SL reads using *C. elegans* direct RNA sequencing datasets.

We classified SL sequences by type using the high-confidence SL-carrying reads identified by SLRanger. Our findings are consistent with the SL1 and SL2 distribution, with SL1 accounting for around 75% of SL-containing reads in all *C. elegans* datasets analyzed (Fig. 3a). The observed lower SL1 ratio in the *C. elegans* YA stage data from Roach *et al*. may be attributed to reduced accuracy stemming from the old basecalling model (Supplementary Fig. S1).

The classification performed by SLRanger clearly distinguished SL1 from SL2 types. Among all high-confidence SL-carrying reads, only 0.05% of reads could not be distinguished between SL1 and SL2 types (Fig. 3b). There were around 9% of reads that could not be clearly assigned between SL2 variants, labeled as “SL2_unknown” (Fig. 3b).

Interestingly, we observed that SL10, a variant of SL2-type, was more abundant than the canonical SL2 variant in certain samples (Fig. 3b), suggesting potential stage-specific or condition-specific SL usage preference.

### SLRanger enabled accurate and sensitive prediction of *C. elegans* operons

Following the SL type assignment for each read using SLRanger, we further utilized this information to infer operon structures. For all the direct RNA sequencing *C. elegans* datasets, SLRanger identified the putative operons and their associated genes (Fig. 3c). To evaluate the prediction performance, we compared the predicted operon genes and structures with the current operon annotation of *C. elegans*, which we treated as the ground truth for evaluation purposes.

Overall, 81% of the predicted operon genes overlapped with the reference annotation, corresponding to a precision of 81%. Meanwhile, 59% of the annotated operon genes were successfully recovered, yielding 73% and 53% sensitivities for the Li *et al*. and Roach *et al*. datasets, respectively. At the structure level, 52% of the predicted operon structures were fully concordant with the ground truth, indicating structure-level precisions of 59% and 48%, and structure-level sensitivities of 51% and 33% for the Li *et al*. and Roach *et al*. datasets, respectively. These results collectively demonstrate that SLRanger not only improves SL sequence detection but also enables reliable operon structure reconstruction in *C. elegans*.

While the overall gene-level precision from the Roach *et al*. dataset was comparable to that of Li *et al*., the Li *et al*. dataset achieved ~20% higher sensitivity for both gene- and structure-level predictions. This difference was also probably due to the insufficient accuracy of the RNA reads from Roach *et al*. Under the same filtering criteria for high-confidence SL sequence reads, fewer reads were retained. Consequently, fewer genes could be covered. Nonetheless, SLRanger maintained the prediction precision (above 80%) even with RNA reads with higher error rates, demonstrating its robustness. Users may fine-tune the dynamic cutoff to achieve optimal performance.

To demonstrate the high resolution of SLRanger, we also used high-confidence SL reads identified by the SW score method to predict operons and compare the recall and precision between the two methods. Our results show that while the accuracy of SLRanger predictions is lower than that of SW score-based predictions, SLRanger achieves a higher overall recall (Fig. 3d and e). Due to the lower resolution of the SW score approach, fewer reads exceed the same cutoff threshold, inflating precision. Our reference true positive set consists only of rigorously verified operons, making it relatively small and inherently a subset of all true operons. In this context, recall is the more informative metric: it shows how effectively each method recovers the validated operons and underscores SLRanger’s capacity to detect additional true operons beyond the reference set. Consequently, SLRanger offers a greater potential for detecting more operons. Users who favor higher precision can simply raise the score threshold to achieve the desired balance between recall and precision.

## Discussion and conclusion

In summary, we developed a novel tool, SLRanger, capable of detecting SL sequences directly from long RNA reads. SLRanger performs SL detection with specific modifications from original SW scores and demonstrates higher resolution than only using conventional SW-based mapping. We validated our approach using datasets from the nanopore direct RNA sequencing and cDNA data of the model organism *C. elegans*, as well as nanopore direct RNA sequencing of *B. malayi*. Although SLRanger was originally developed for the ONT direct RNA sequencing platform, the pipeline is also applicable to other long-read cDNA sequencing platforms. We have demonstrated SLRanger’s performance on ONT cDNA sequencing datasets with an average *Q* score of 10 with optimized parameters. Moreover, we expect SLRanger to perform well on higher-accuracy cDNA sequencing platforms, including PacBio Iso-Seq. Because cDNA synthesis involves reverse transcription and amplification, it introduces more soft clipping at the 5′ end of reads. Misplacement of soft-clipped regions during mapping to the reference genome may lead to the loss of SL information in some reads. Besides, Bernard *et al*. predicted that the SL sequence could form a hairpin structure with an unpaired overhang of ~9 nt and observed that non-trans-spliced mRNAs can form hairpin structures when analyzing *C. elegans* cDNA data [31]. However, we didn’t detect such hairpin signals in the 5′ soft-clipped regions of direct RNA sequencing data (Supplementary Fig. S3). Direct RNA sequencing can only capture the linearized, single-stranded RNA, rather than the folded secondary structures. So the length distribution of detected SL residual (<9 nt) (Fig. 2d) may indirectly support Bernard’s cDNA-based finding that SL sequences can form hairpin structures at the 5′ end.

SLRanger also provides the function to predict operons, leveraging the subtyping of SL, the fusion reads detection, and the locations of adjacent genes. Across all datasets, our predictions achieved an average accuracy exceeding 80%. The remaining 20% may represent potential operon candidates, but the status requires further validation. We have also compiled the genes that do not appear in the annotated operons into a supplementary table, which is available at the Figshare supplementary link. Among the genes detected by SLRanger but not included in the annotated operon genes, ~20% belong to operons where other constituent genes are already covered by the annotated operon combinations. For the remaining genes, we are currently unable to determine their authenticity. It is possible that some may be false positives, and further validation is needed. The high recall and precision of the predicted operon also prove the authenticity of the SL reads detected by SLRanger.

Apart from the SL sequences we identified, many 5′ unaligned reads also exhibit soft clipping, which may reflect biologically meaningful events rather than technical issues alone. Possible biological causes include the diversity of TSSs [44], unannotated 5′ exons, and alternative promoters [45]. We have not yet conducted a comprehensive analysis of these unaligned 5′ regions beyond SL detection.

Overall, SLRanger not only introduces a new computational strategy for detecting SL events and predicting operon structures, but also contributes a practical pipeline to support research in SL trans-splicing. Unlike previous approaches that largely relied on short-read sequencing data or PCR-based amplification of known SL sequences, SLRanger leverages native long-read RNA sequencing and a refined scoring scheme to enable transcriptome-wide detection of SL events. Importantly, SLRanger opens new avenues for systematic SL discovery in eukaryotes and operon annotation in nematodes, including those with limited genomic annotations. Furthermore, by enabling high-resolution characterization of SL trans-splicing at the transcriptome-wide scale, this tool facilitates broader opportunities for data-mining and functional transcriptomic exploration, thereby contributing to deeper insights into gene regulation, operon organization, and post-transcriptional mechanisms in complex genomes.

**Key Points**

- Spliced leader (SL) trans-splicing plays a critical role in nematodes and diverse microbial eukaryotes and participates in proper mRNA processing from operons.
- Long-read RNA sequencing technologies, such as direct RNA sequencing by Oxford Nanopore Technologies, offer a more promising avenue for detecting these events with greater resolution than short-read sequencing.
- We introduce SLRanger, an integrated tool to detect SL sequences and predict operon structures in eukaryotic transcriptomes.
- Validated in the model organism *Caenorhabditis elegans*, SLRanger reliably detected high-confidence SL-carrying reads and achieved precise operon prediction.

## Supplementary Material

Supplementary_figures_bbaf437

TableS1_bbaf437

## Data Availability

Data processed were submitted to the NCBI under BioProject PRJNA1254851. SLRanger is available on Github (https://github.com/lrslab/SLRanger). All other supplementary files were deposited in Figshare (https://doi.org/10.6084/m9.figshare.29570606).
