# Supplementary material for: SLRanger: an integrated approach for spliced leader detection and operon prediction using long RNA reads
Source: Brief Bioinform. 2025 Sep 4;26(5):bbaf437. doi: 10.1093/bib/bbaf437 (PMC12410068; doi:10.1093/bib/bbaf437)
Supplement: Supplementary_figures_bbaf437 [file supplementary_figures_bbaf437.pdf]

Supplementary figures

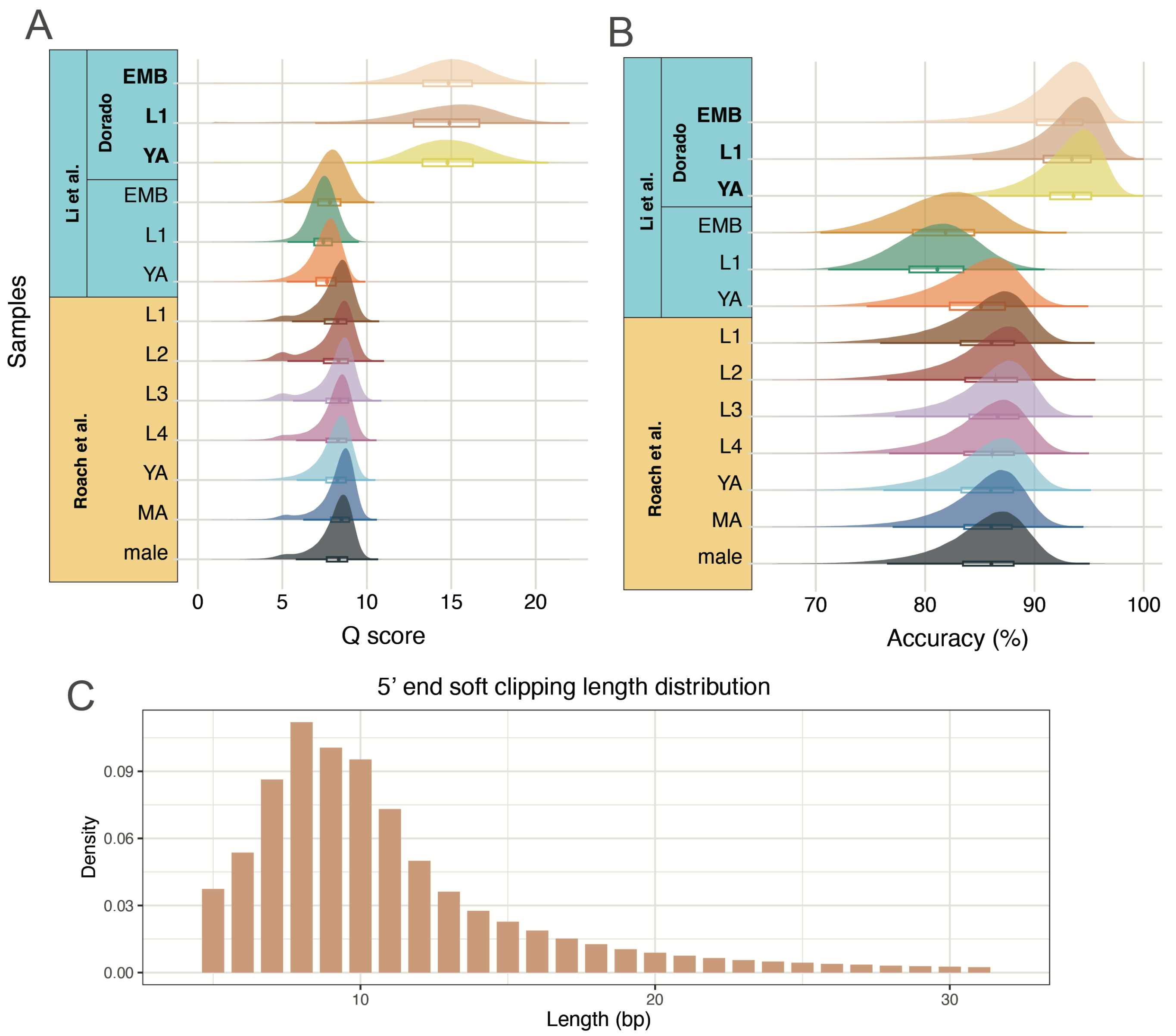

Figure S1. Quality controls of the used *C. elegans* direct RNA sequencing dataset. (A) Density plots of estimated Q scores of all used datasets. The average Q scores of the dorado basecalled datasets from Li et al. were higher than those of others. (B) Density plots of the mapping observed accuracy of all used datasets. The average mapping observed accuracies of the dorado basecalled datasets from Li et al. were higher than those of others. (C) Soft length density distribution of the average 5’ end soft clipping length.

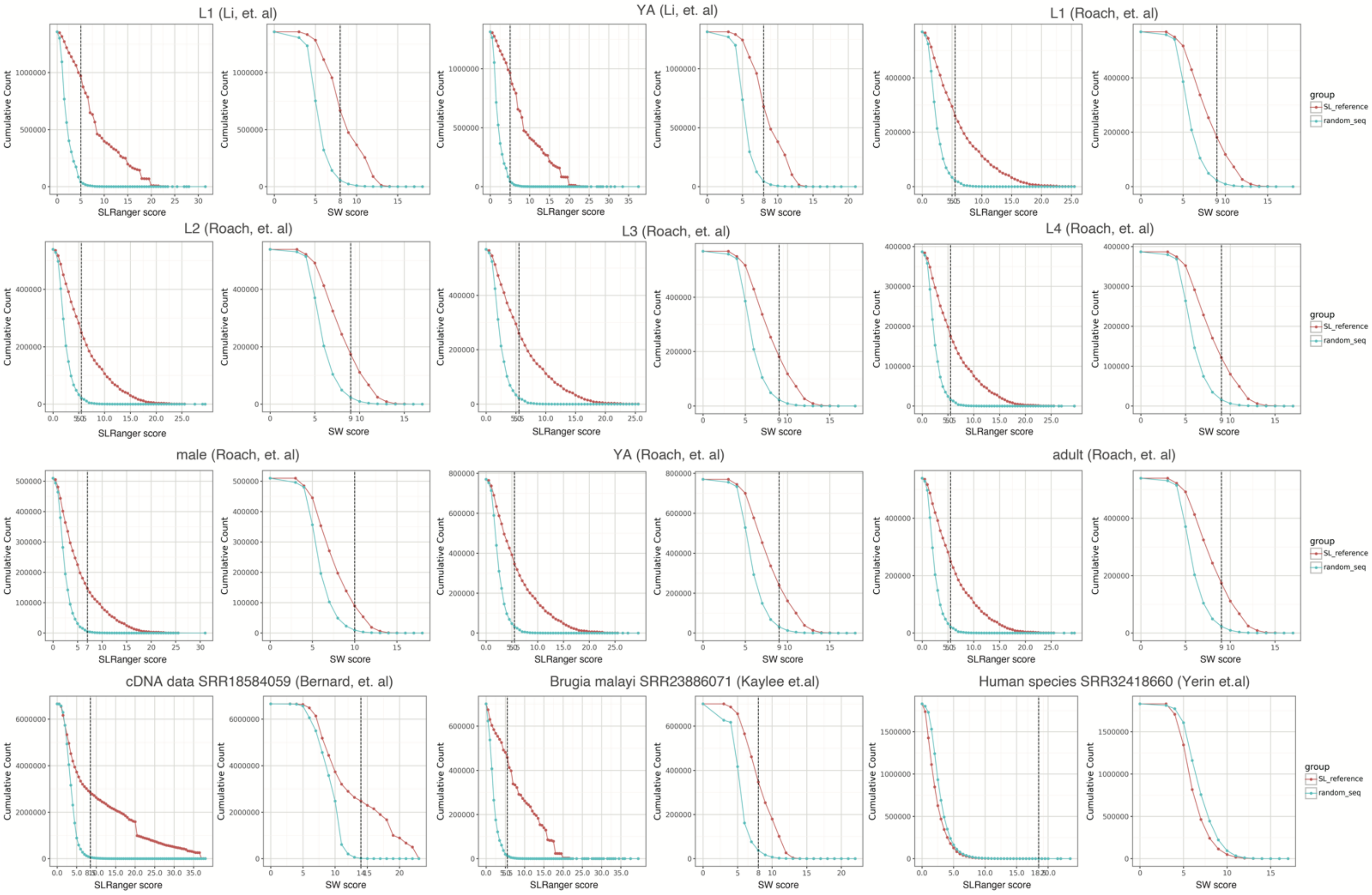

Figure S2. The AUC plots illustrate the dynamic thresholding performance of SLRanger score and Smith-Waterman (SW) alignment optimal score of all other datasets.

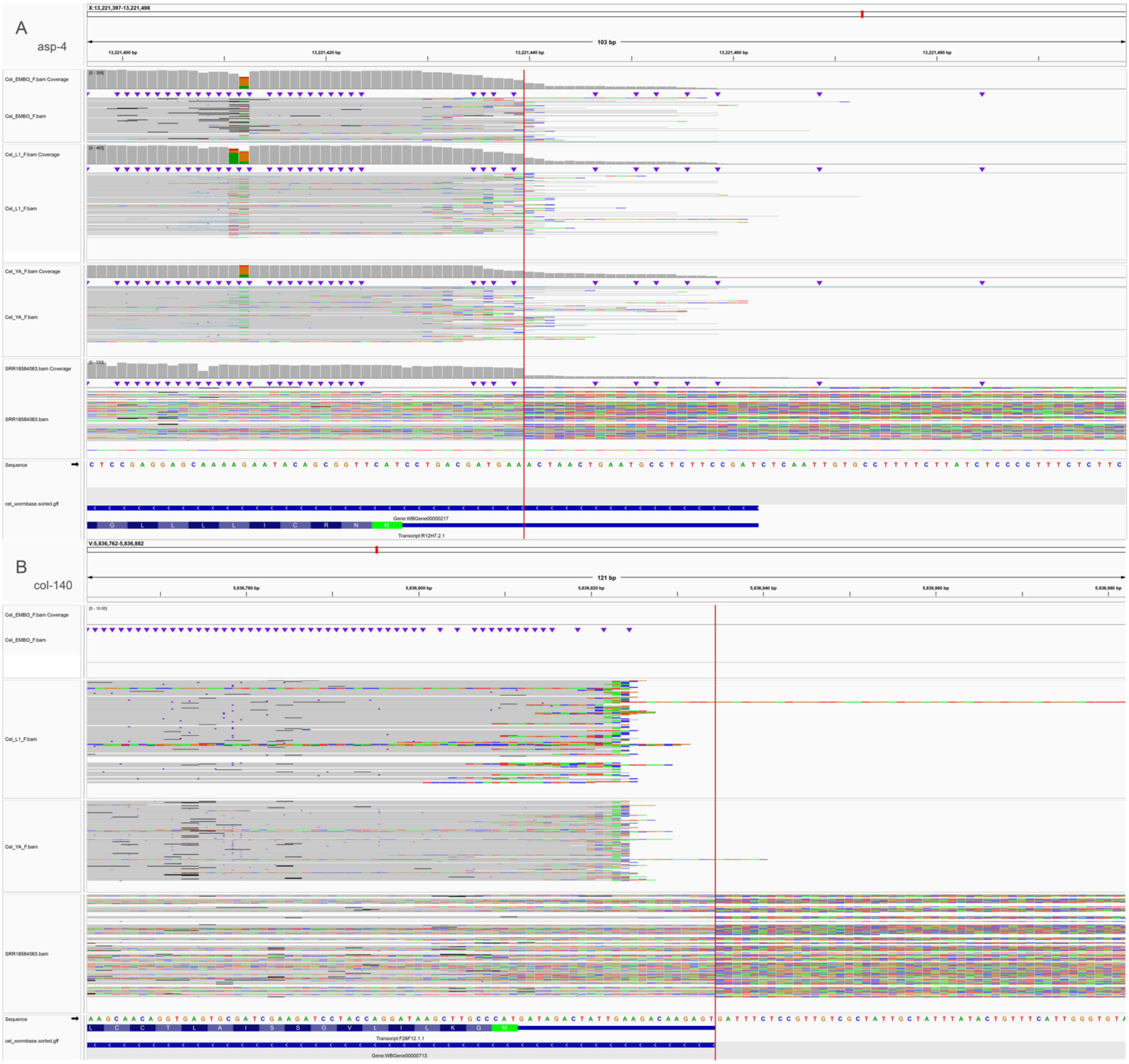

Figure S3. Comparison of Li et al. Direct RNA Sequencing and Bernard et al. cDNA data in IGV views of asp-4 (A) and col-140 (B), whose mRNA was reported to have a hairpin structure at the 5' end. The top three tracks show direct RNA sequencing data from Li et al., while the fourth track displays cDNA data from Bernard et al. Notably, the direct RNA sequencing data do not exhibit the soft-clipping regions characteristic of the 5' stem-loop structures reported by Bernard et al., which are evident in the cDNA dataset.
